# Eruption of Generalized Pustular Psoriasis Following Initiation of Adalimumab for Hidradenitis Suppurativa: A Case Report

**DOI:** 10.7759/cureus.75402

**Published:** 2024-12-09

**Authors:** Christopher D Markeson, Simo Huang, Sylvia Hsu

**Affiliations:** 1 Dermatology, Lewis Katz School of Medicine, Temple University, Philadelphia, USA

**Keywords:** biologic, drug reaction, generalized pustular psoriasis, paradoxical psoriasis, tumor necrosis factor

## Abstract

Rarely, tumor necrosis factor (TNF)-α inhibitors can paradoxically induce eruptions of psoriasis with generalized pustular psoriasis being among the least common presentations. We report a patient who presented with a generalized pustular eruption following adalimumab therapy for hidradenitis suppurativa (HS). The diagnosis of generalized pustular psoriasis was confirmed with a biopsy showing neutrophilic spongiosis and intraepidermal pustulosis. We discuss the role of tumor necrosis factor in psoriasis while describing possible mechanisms for this paradoxical reaction. Additionally, we discuss treatment options while considering the severity of the eruption and the underlying disease being treated with the tumor necrosis factor inhibitor.

## Introduction

Adalimumab is a human IgG monoclonal antibody targeting and neutralizing tumor necrosis factor (TNF) activity [1]. It is used in the treatment of various inflammatory dermatoses, including psoriasis and hidradenitis suppurativa (HS). In patients with HS, adalimumab reduces the secretion of cytokines IL-1β, CXCL9, and B-lymphocyte chemoattractant (BCL) along with CD11c+, CD14+ and CD68+ cells [2]. Only 2-5% of patients receiving TNF inhibitors for other conditions, such as rheumatoid arthritis or inflammatory bowel disease, develop paradoxical psoriasis with generalized pustular psoriasis being among the least common presentations [3]. 

## Case presentation

**Figure 1:**
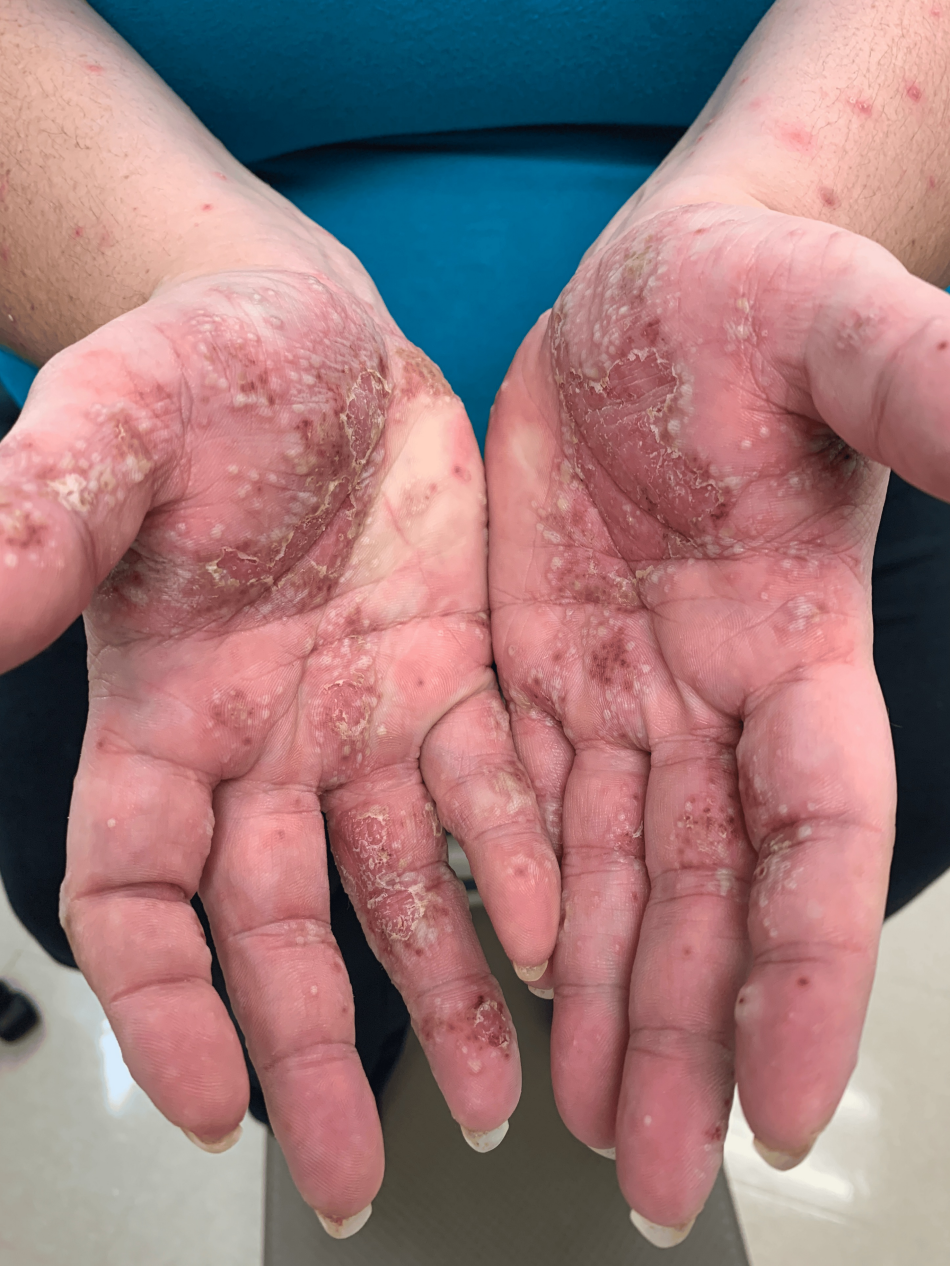
Palms and arms with pustules on an erythematous base and scaly plaques.

**Figure 2:**
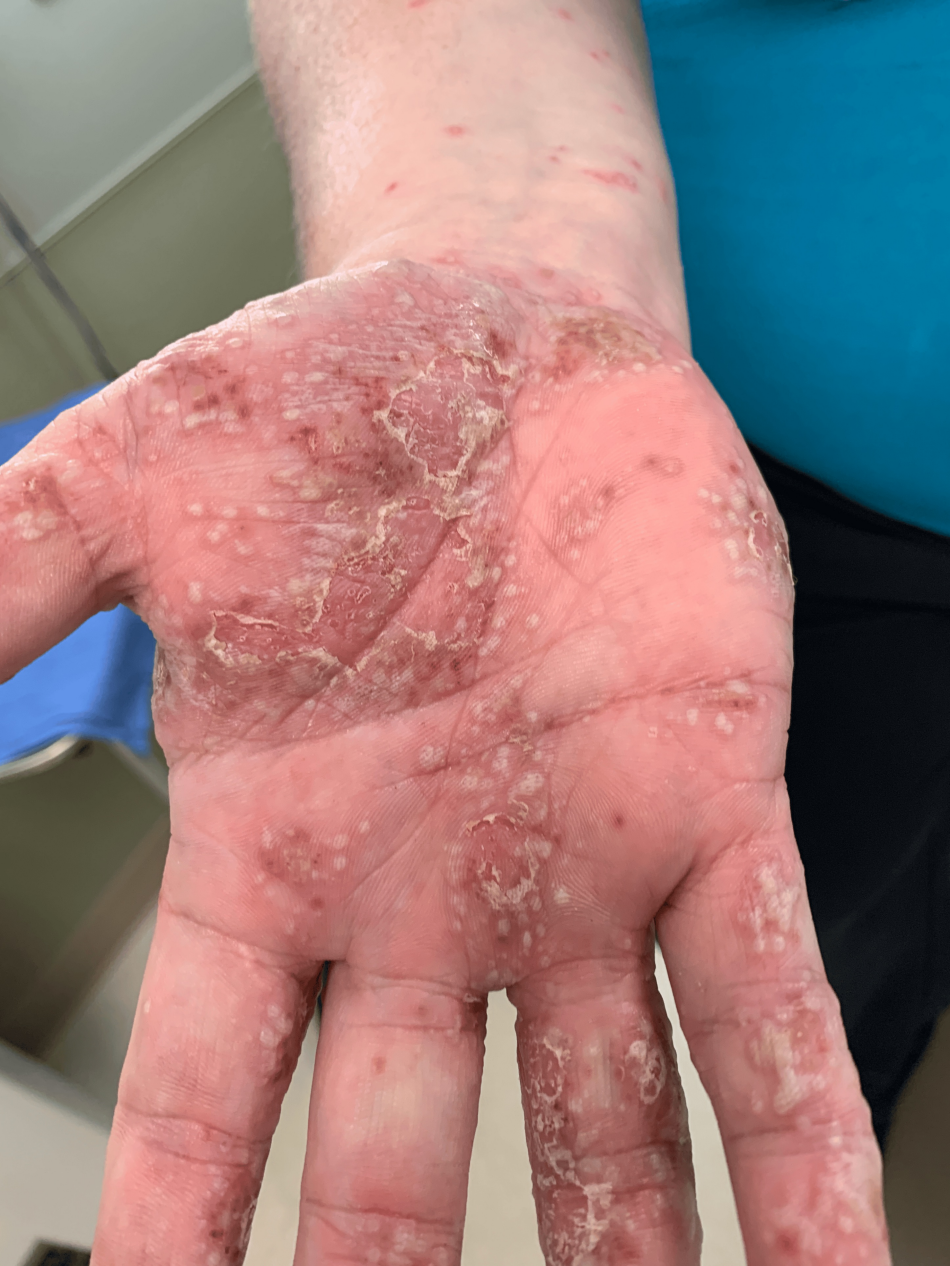
On the right palm and ventral forearm, there are numerous pustules on an erythematous base.

**Figure 3:**
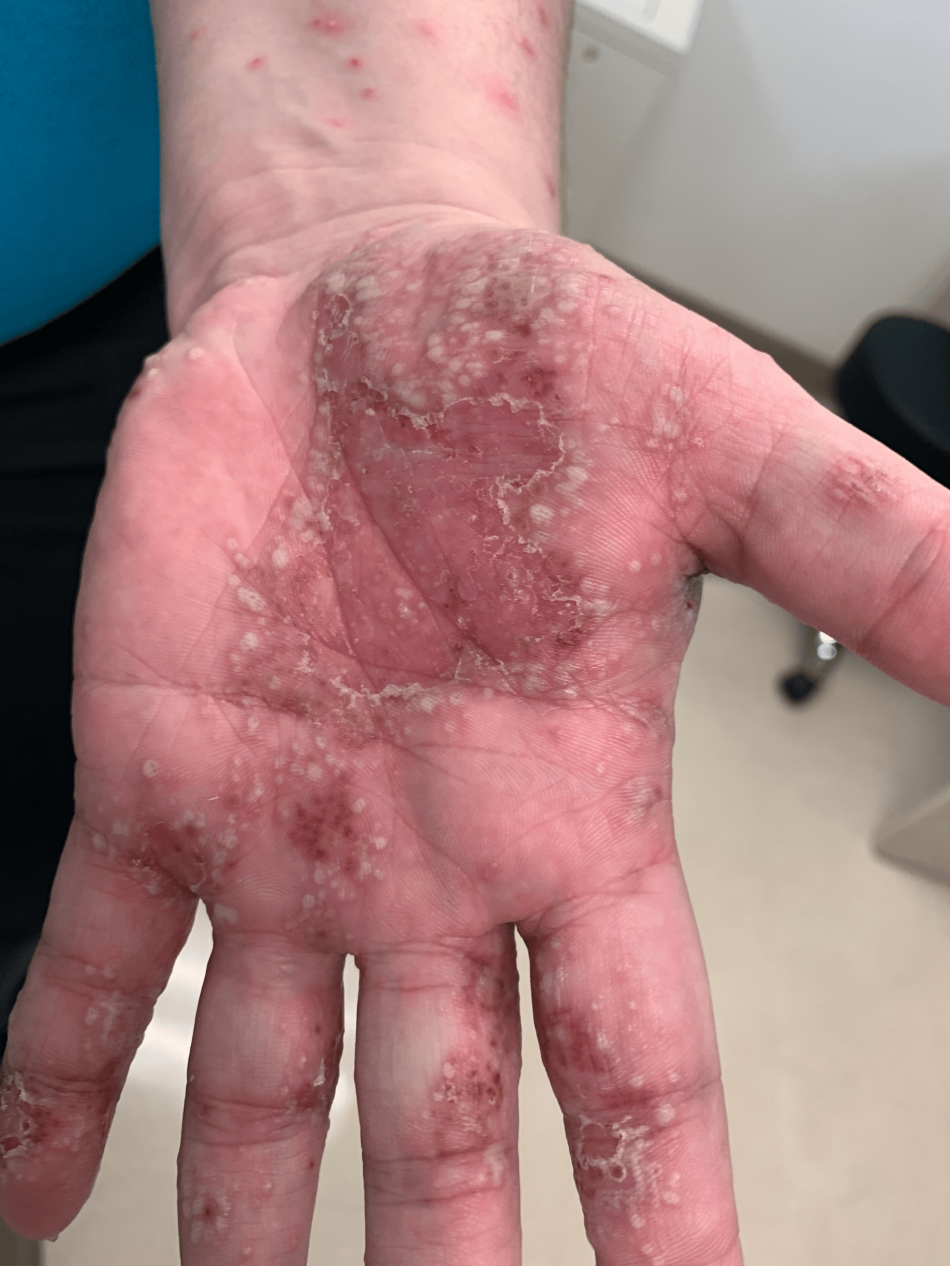
On the left palm and ventral forearm, there are numerous pustules on an erythematous base.

**Figure 4:**
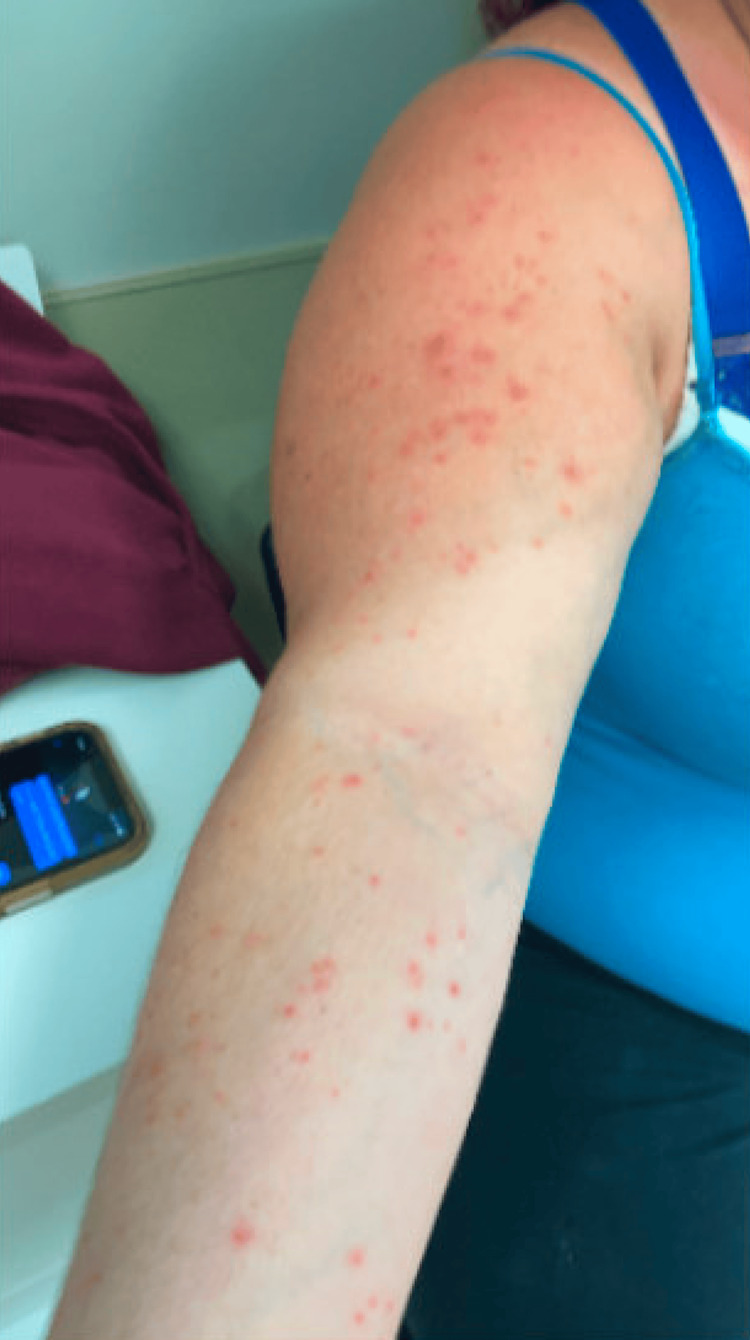
On the right arm, there are numerous pustules on an erythematous base.

**Figure 5:**
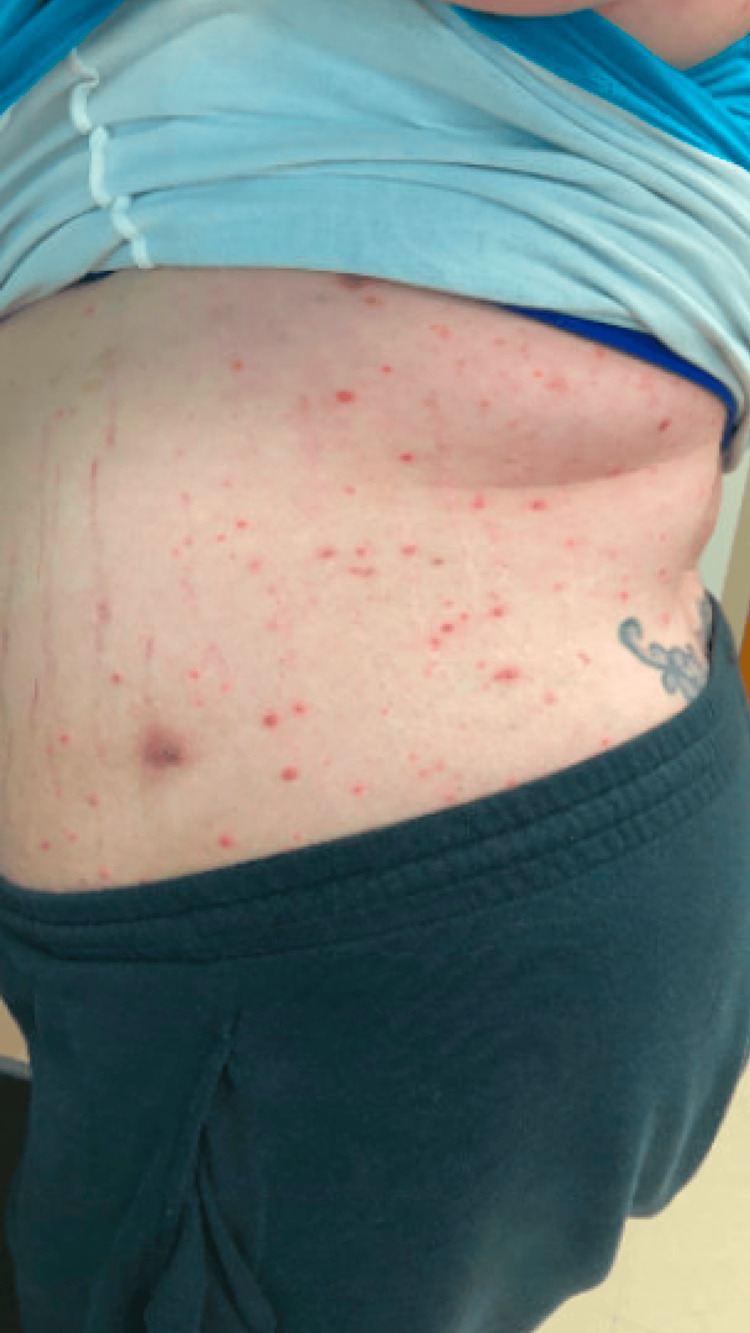
On the trunk, there were numerous pustules on an erythematous base.

A 34-year-old Caucasian woman with a 20-year history of hidradenitis suppurativa (HS) involving her axillae, inframammary folds, and groin (previous treatments included benzoyl peroxide, clindamycin gel, bleach baths, doxycycline, oral clindamycin, and intralesional triamcinolone) presented with joint pain (of her elbows, wrists, hips, knees, and ankles) and a diffuse rash two months after starting adalimumab for her HS. She had no personal or family history of psoriasis, and she was a heavy smoker. The painful and pruritic rash started on her palms and progressed centripetally. Physical examination revealed numerous pustules and collarettes of scale on an erythematous base on the palmoplantar areas (Figures 1, 2, 3), arms (Figure 4), legs, and trunk (Figure 5).

**Figure 6:**
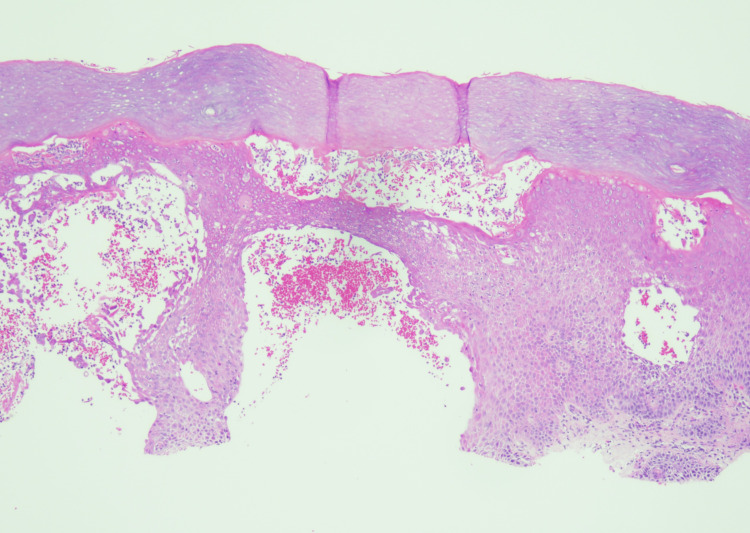
Biopsy of a pustule on left palm. Intraepidermal and subcorneal pustules with neutrophilic spongiosis on acral skin consistent with pustular psoriasis (H&E, 40x).

**Figure 7:**
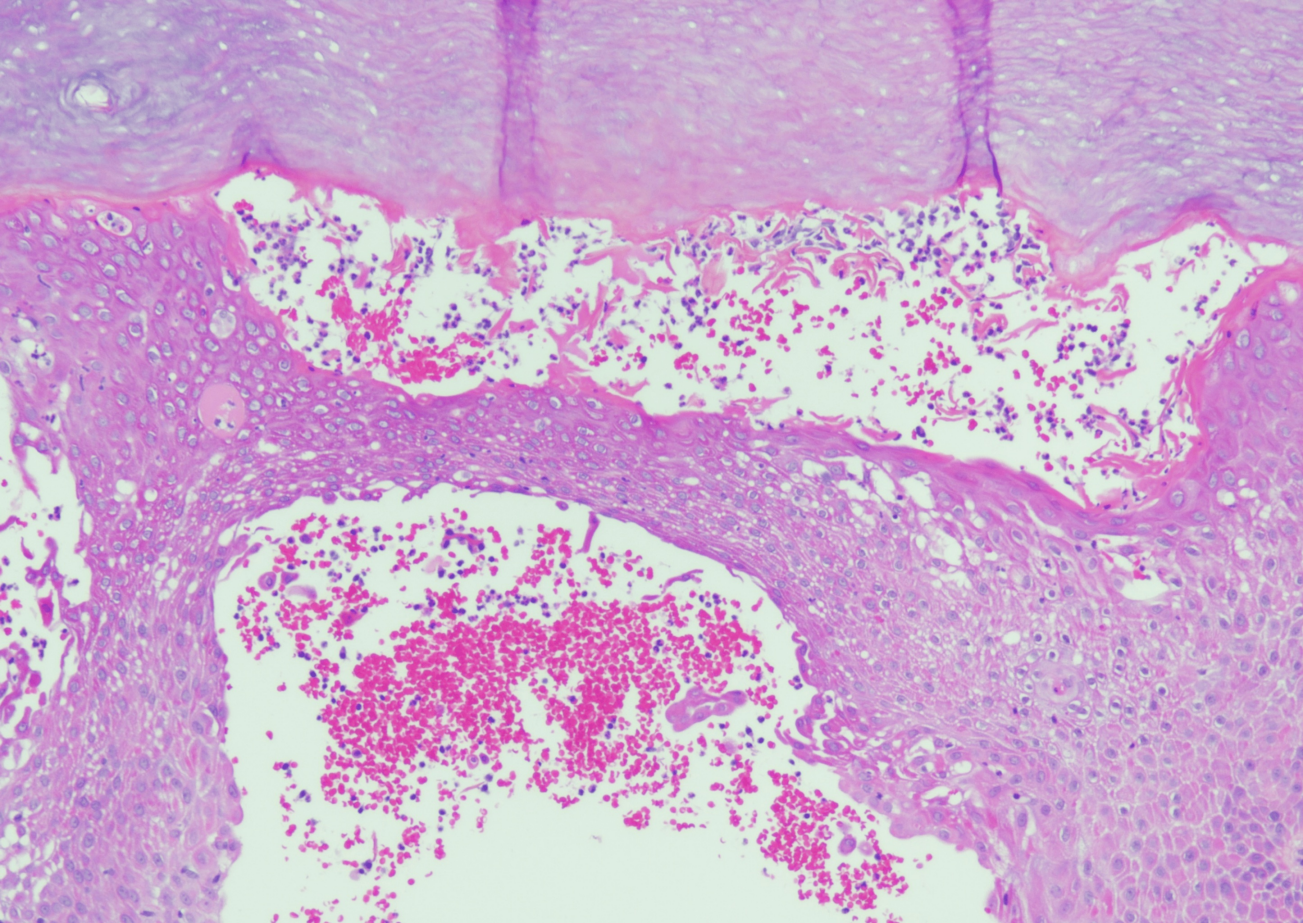
Biopsy of a pustule on the left palm. Intraepidermal and subcorneal pustules with neutrophilic spongiosis on acral skin consistent with pustular psoriasis (H&E, 100x).

A shave biopsy of an acral lesion showed subcorneal pustules (Figures 6, 7) and diffuse hypogranulosis, consistent with pustular psoriasis. The histopathological differential diagnosis included acute generalized exanthematous pustulosis. 

Given the clinical findings, the latency period of the onset of the generalized pustules, and the culprit drug, the patient was diagnosed with pustular psoriasis at the initial visit and was prompted to continue her adalimumab for HS, since it was improving her HS, and start cyclosporine 300 mg divided into two doses daily for her generalized pustular psoriasis. She discontinued adalimumab therapy one week prior to her follow-up three weeks following the initial presentation. Due to worsening cutaneous involvement and concurrent joint pain, she was started on guselkumab, while continuing her cyclosporine. On the second follow-up two months following the initial presentation, the patient had improvement in her GPP and was instructed to begin tapering off cyclosporine.

## Discussion

The exact mechanism by which TNF inhibitors induce psoriatic eruptions is unknown, but several pathways are hypothesized to play a role. TNF-α normally attenuates plasmacytoid dendritic cells by inducing cellular maturation and ceasing the production of interferon-α. Inhibition of TNF-α leads to unregulated production of interferon-α, a potent stimulant for T helper type 1 (Th1) lymphocytes. In the acute phase of classical psoriasis, interferon-α from plasmacytoid dendritic cells activate conventional dendritic cells responsible for the production of TNF-α and IL-23. In the chronic phase of classical psoriasis, TNF-α and IL-23 activate potentially autoreactive CD8+ T cells while forming a negative feedback loop with interferon-α production. This shifts the main driver of pathogenicity from interferon-α regulation to T cell-mediated with CD8+ T cells in the epidermis releasing IL-17 and IL-23 and causing keratinocyte hyperproliferation [3]. Conversely, psoriasis caused by TNF inhibition displays characteristics more in common with the early phase of classical psoriasis. In the setting of decreased TNF-α, conventional dendritic cells are not able to activate the CD8+ T cells and cannot perform negative feedback on interferon-α. This causes an interferon-driven acute-phase immunologic reaction independent of T-cell activation [3]. Patients with TNF inhibitor-induced psoriasis show increased interferon-α expression in the dermal vasculature of their lesions [4]. 

Proposed treatments for patients with TNF inhibitor-induced psoriasis depend on the severity of the eruption, the underlying condition being treated, and the form of psoriasis eruption [5,6]. In mild cases and controlled underlying disease, a “treat through” approach includes the continuation of the TNF inhibitor along with additional psoriasis treatments, such as topical corticosteroids, ultraviolet light therapy, and cyclosporine [7]. Additionally, treatment with dapsone and acitretin has been successfully used in patients with pustular psoriasis and can be considered when patients have eruptions induced by TNF inhibitors [7,8]. When patients have either uncontrolled underlying disease with mild psoriatic involvement or controlled disease with moderate-to-severe psoriatic involvement, recommendations have been made to switch to a different TNF inhibitor while starting psoriasis-targeted therapy. In uncontrolled underlying disease and moderate-to-severe psoriatic involvement, patients should discontinue the TNF-α inhibitor and switch to a different drug class, such as an IL-17 inhibitor (secukinumab) for treatment [8,9]. The average time to onset of lesions was 14 months after the initiation of the TNF inhibitor and did not show significant variation depending on the underlying condition being treated [3,5,6]. Patients with generalized pustular psoriasis showed the greatest rates of improvement or resolution of disease when the anti-TNF therapy was switched or discontinued [3].

## Conclusions

The true mechanism of psoriatic eruptions following TNF inhibitor initiation is not fully understood and can complicate treatment in patients who need biologic treatment for a separate diagnosis. We present this case to report on a rare reaction of generalized pustular psoriasis secondary to the initiation of adalimumab for HS. One should consider the effects on both the primary diagnosis and the secondary psoriatic eruption when deciding to “treat through,” adding ancillary treatments or switching systemic therapy.
